# The core outcome set for studies on feminizing genital gender-affirming surgery: findings from the GenderCOS project

**DOI:** 10.1016/j.eclinm.2025.103323

**Published:** 2025-07-01

**Authors:** Marleen S. Vallinga, Philippine J. Roijer, Thomas E. Pidgeon, Matteo Angelini, Aline Ceulemans, Alex Bakker, Brenda Carrière, Tina Rashid, James Bellringer, Javier Belinky, Marlon Buncamper, Shane D. Morrison, Walter P. Bouman, Tim C. van de Grift, Mark-Bram Bouman, Margriet G. Mullender

**Affiliations:** a Department of Plastic, Reconstructive and Hand Surgery, Amsterdam UMC (Location VUmc) - Amsterdam University Medical Centre, Amsterdam, the Netherlands; b Amsterdam Public Health Research Institute, Amsterdam, the Netherlands; c Department of Plastic Surgery, Russells Hall Hospital, Dudley Group Foundation Trust, Dudley, United Kingdom; d IRCCS San Raffaele Hospital, Vita-Salute San Raffaele University, Milan, Italy; e Department of Plastic Surgery, University Hospital Ghent, Ghent, Belgium; f Independent Scholar, Utrecht/’s Hertogenbosch, the Netherlands; g Chelsea Centre for Gender Surgery (CCGS), Chelsea and Westminster Hospital NHS Trust, London, United Kingdom; h Nuffield Health Parkside Hospital, London, United Kingdom; i Guemes Hospital and Urological Center CDU, Buenos Aires, Argentina; j Division of Plastic and Reconstructive Surgery, Department of Surgery, University of Washington, Seattle, WA, United States of America; k Department of Urology, University of Washington, Seattle, WA, United States of America; l Nottingham Centre for Transgender Health, Nottingham, United Kingdom; m Department of Psychiatry and Medical Psychology, Zaans Medical Center, Zaandam, the Netherlands

**Keywords:** Gender-affirming surgery, Core outcome set, Genital surgery, Transgender, Feminizing

## Abstract

**Background:**

Feminizing genital gender-affirming surgery (gGAS) comprises various surgical procedures and techniques. Comprehensive knowledge of the outcomes of all procedures and techniques is fundamental for informed decision-making. However, in current research on feminizing gGAS there is significant heterogeneity in reported outcomes. Standardization of outcome measurement is therefore urgently required. This study aimed to develop a Core Outcome Set (COS) for feminizing gGAS.

**Methods:**

A multidisciplinary, international study steering group comprising 16 panellists from Europe, the United Kingdom, North America, and South America—including health care professionals and transgender individuals—was appointed to guide the development of a COS. The steering group convened on 16 June 2022, 28 September 2023, 2 May 2024, and 29 August 2024. The study involved three phases: (i) relevant outcomes were identified through a literature review and focus groups with transgender and gender-diverse people; (ii) stakeholders were invited to participate in an international Delphi study to reach consensus on the key outcomes; (iii) a consensus meeting was held to reach a final consensus on the COS.

**Findings:**

Initial data collection yielded 2621 unique outcomes. This number was reduced to 39 potential outcomes for the Delphi process through a structured selection process. The Delphi rounds encompassed February, May and July 2024 respectively. Following the consensus meeting in September 2024, a final list of 11 outcomes was agreed, of which six are patient-reported outcomes. Seven outcomes apply to all feminizing gGAS procedures and four are specific to vaginoplasty procedures involving the creation of a vaginal canal.

**Interpretation:**

Adoption of the COS for feminizing gGAS could ensure that the most relevant outcomes in clinical research are measured and reported in a standardized way. Future studies adopting these suggested outcome measures could reduce the heterogeneity of reported outcomes across studies and working to improve the quality of research and care.

**Funding:**

None received.

**Research in context**

**Evidence before this study**

In 2022, the Core Outcome Measures in Effectiveness Trials (COMET) registry and PubMed were searched for existing studies or initiatives to develop a Core Outcome Set (COS) for genital gender-affirming surgery (gGAS), which yielded no results. Several reviews were identified that reported on the diversity, lack of outcome standardization, and low-quality studies on feminizing gGAS procedures. One comprehensive review on outcome reporting in studies on feminizing gGAS was identified, published as a conference abstract. The authors of this review were approached, and the gathered data from the review was shared for the purpose of this study.

**Added value of this study**

Through an extensive consensus procedure, this study has identified outcomes that are considered of critical importance for research on feminizing gGAS, as determined by both members of the target population and clinical experts in the field. The developed COS facilitates the standardization of outcome selection for studies on gender-affirming surgical care involving vaginoplasty, vulvoplasty, clitoroplasty, and labiaplasty, whether as primary or revision procedures. The final COS includes both patient-reported and clinical outcomes, providing a foundation for future research, guideline development, and informed decision-making.

**Implications of all the available evidence**

This consensus study for the development of a COS for feminizing gGAS is a significant step towards standardizing outcome research in this field. Involving lived experience experts in this process was instrumental in ensuring patient-centeredness. The next step is to establish standardized methods for measuring and reporting these core outcomes. Once the COS has been widely adopted in feminizing gGAS research, meaningful conclusions can be drawn about outcomes that are relevant to the population concerned and experts in the field.

## Introduction

Transgender and gender-diverse individuals often seek to align their physical traits with their gender identity through gender-affirming treatments, including hormonal and surgical interventions.^1^^,^^2^ Among these treatments, surgical alignment of the genital area is of vital importance to many, as it enhances gender congruence and can significantly improve quality of life.^3^^,^^4^

Feminizing genital gender-affirming surgery (gGAS) involves various procedures. Most individuals seeking feminizing gGAS desire the creation of a vulva, including clitoris and labia, and a full-depth vaginal canal, i.e., vulvo- and vaginoplasty. However, as awareness of gender diversity increases and individualized treatment goals are more widely discussed, shallow or no-depth vulvoplasty is becoming more common.^5^ Reported reasons for vulvoplasty include patient preferences concerning gender-affirmation, sexual function, reluctance to commit to life-long vaginal dilatation, a lower risk of complications, and medical recommendation by the surgeon.^5^^,^^6^ Several technical variations have been described for both surgical procedures. However, the greatest variation in surgical technique is seen in the creation of the vaginal canal, where there are several operative approaches, many technical variations, and different lining tissues being employed.^7^ Indications for techniques vary depending on the availability of the donor tissue, but are often based on surgeon preference. Transparent, relevant, and evidence-based information about the available surgical options should be available to both the care seeker and the surgeon to enable shared, informed decision-making about the procedure and technique.^2^

The current scientific evidence on feminizing gGAS is of insufficient quality to allow for evidence-based shared decision making. The scientific literature on feminizing gGAS is primarily composed of single-center retrospective case series, with a focus on surgical outcomes and the safety of one specific technique. There is a lack of long-term outcomes, including patient-reported outcomes (PROs), which are crucial for evaluating patient experiences regarding satisfaction, gender congruence, sexual and psycho-social well-being.^8^, ^9^, ^10^ There is a need for well-designed prospective clinical research. Heterogeneous outcome reporting and a lack of population involvement in research are also hindering progress in this field.^11^ The research community has yet to establish consensus on which outcomes to measure, as well as ‘how’ and ‘when’ to measure them, and these gaps collectively impede the comparison of results and the synthesis of meaningful data.^8^^,^^12^

To address this deficit, the aim of this study is to develop an international multi-stakeholder consensus-based Core Outcome Set (COS) for feminizing gGAS. COSs are recognized as a means to improve the quality and relevance of clinical research.^13^ They are an agreed, standardized set of outcomes that should, at minimum, be measured and reported in all clinical research in the specific health(care) area concerned.^14^ By engaging clinical experts and population representatives in the development process, the COS addresses the lack of standardization and evidence gaps while ensuring the relevance of research to those undergoing these surgical treatments.

## Methods

### Study overview

**Fig. 1:**
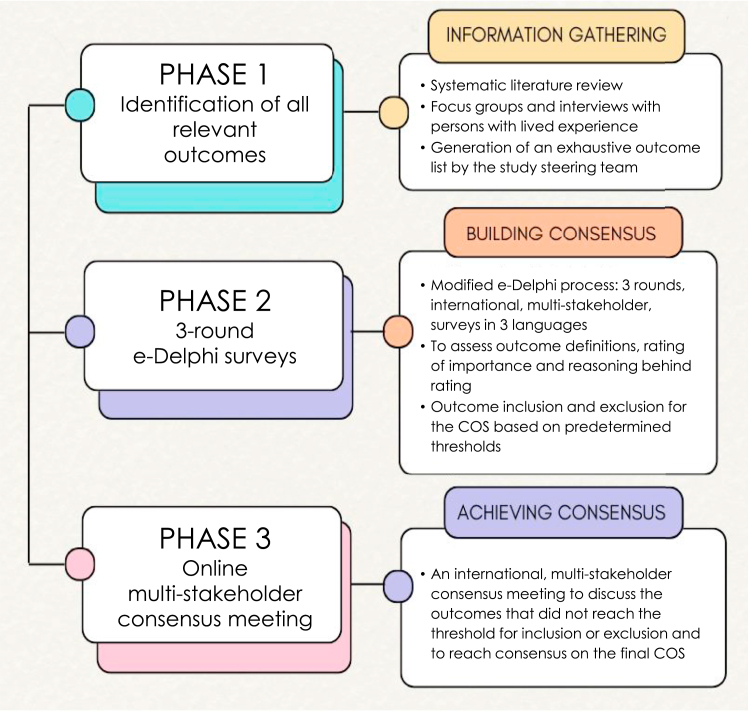
Overview of the GenderCOS development phases.

The GenderCOS project involves the development of two COSs: one for feminizing gGAS and one for masculinizing gGAS. This article describes the development of a COS for feminizing gGAS, which followed the recommendations of the Core Outcome Measures in Effectiveness Trials (COMET) Handbook.^14^ The project started in September 2021. First, an international, multidisciplinary study steering group (SSG) was formed comprising 16 panellists from Europe, the United Kingdom, North America, and South America. Panellists were invited based on their expertise in gGAS and research, geographical location, and willingness to invest time in the project. Two independent lived experience experts were also invited. All steering group members are listed as authors on the manuscript. The SSG was involved in all project stages, including defining the scope and the study design. The steering group convened on 16 June 2022, 28 September 2023, 2 May 2024, and 29 August 2024. Between these meetings, the SSG received regular updates and communication via email. The study protocol was published prior to the initiation of the Delphi process.^15^ The study comprised three phases: 1) the identification of all relevant outcomes, 2) a Delphi procedure to reach a consensus on the most important outcomes, and 3) a consensus meeting to reach an agreement on the final COS (Fig. 1). The COS was established in September 2024. Detailed information about the study methods can be found in the Supplemental materials. This article adheres to the COS Standards for Reporting statement (COS-STAR).^16^

### Scope of the feminizing gGAS COS

The scope of this COS is described in the study protocol according to the recommendations of the COS Standards for Development (COS-STAD).^15^^,^^17^ Briefly, the COS for feminizing gGAS applies to clinical research on all surgical procedures for the feminization of the genitalia, i.e., vaginoplasty, vulvoplasty, cliteroplasty, and labiaplasty, either as a primary or revision procedure. The research population concerned is transgender and gender-diverse people who are of legal age for surgery and who choose to undergo genital feminizing surgery as part of their gender-affirming treatment.

### Ethics

The study is registered in the COMET database under study number 2067.^18^ Ethical approval for the previous qualitative study and the GenderCOS study was obtained from the Amsterdam UMC Medical Ethics Committee, VUmc Institutional Review Board (FWA00017598, reference numbers: 2020.0653 and 2021.0026). All participants in the interviews and focus groups gave informed consent for the study and for the data to be used for future research. The transcripts from this previous study were re-analysed for this study.^19^ All participants in the Delphi process gave informed consent prior to the first Delphi survey.

### Participants

In the GenderCOS study, two key stakeholder groups were included: 1) Lived Experience Experts (LEE) and 2) Professional Experts (PE), i.e., researchers and healthcare professionals in genital gender-affirming surgical care. Specific inclusion and exclusion criteria for both stakeholder groups are detailed in the protocol and Supplemental material (S2).^15^ Before and during the first round of the Delphi study, eligible participants could register at the project website, which provided all necessary information about registration and participation. Participants were recruited through social media, the project website, posters at clinics specializing in gender care, emails, and eblasts from the World Professional Association for Transgender Health (WPATH). Additionally, the study protocol was presented at various international conferences in transgender care and sexology, where the registry was actively promoted amongst attendees. Patient advocacy groups worldwide were approached to share promotional materials with their members. All participants were given the opportunity to be acknowledged as contributors, provided they completed all three rounds of the Delphi study.

### Phase 1–identification of potential core outcomes

Potential outcomes were identified through a literature review and qualitative data collection from transgender and gender-diverse individuals. The systematic literature review identified all individual outcomes reported in clinical research on feminizing gGAS.^12^ Before the start of this project, a qualitative study was conducted to inform development of a decision-aid for feminizing gGAS.^19^ In interviews and focus groups, transgender individuals who had chosen or undergone feminizing gGAS were asked what outcomes they considered important in deciding whether to undergo genital surgery and which specific procedures to choose. All outcomes mentioned in the interview and focus group transcripts were identified. The identified outcomes were combined and duplicates were removed, resulting in an exhaustive list of outcomes. First, all verbatim outcomes were sorted and categorized into domains according to Dodds’ taxonomy.^20^ Similar outcomes were deduplicated. This process was repeated to condense similar outcomes. Some ‘outcomes’ were excluded because they were not actual outcomes. The study team then discussed the wording of the aggregated outcomes, as there were often different nuances in their phrasing. A pre-selection of outcomes was necessary to produce a feasible number for the Delphi study. This was done in two steps. The most relevant outcomes were identified through an iterative process. This involved holding several discussions with methodological experts and researchers with experience in COS development. Topics covered included whether complications should be included separately or as a group and the level of granularity of the outcomes. The criteria used to determine relevance included how frequently the outcomes were reported and common sense. The qualitative data were checked to ensure that outcomes identified as important or relevant by transgender and gender diverse individuals were included. The team discussed outcome selection and sought expert opinions iteratively until consensus was reached. In a final step, the study team categorised the remaining outcomes into two groups based on estimated potential to be a core outcome. This was based on input from transgender individuals, clinical relevance and the importance of the outcome in decision-making for feminizing gGAS. The categorization was discussed with independent healthcare professionals to ensure that arguments regarding the relevance of the outcomes had not been overlooked. The categorization was then submitted to the SSG members, who were asked to select potential core outcomes. The survey results were analysed and presented to the SSG in a structured way, showing the categorised outcomes and the SSG members’ ratings. The final selection for the Delphi list was then made using a structured approach in which all SSG members were asked to provide feedback on the proposed list and suggest discussion points. All points were then discussed, resulting in consensus being reached on the final Delphi list. A proposed definition and lay language explanation were formulated for each of these outcomes, which were translated from English into Dutch and Spanish in accordance with the protocol.^15^

### Phase 2–Delphi procedure

A modified Delphi procedure was conducted in three rounds of online surveys using the LimeSurvey platform (LimeSurvey GmbH, n.d.). Participants could only progress to the next round if they had completed the preceding ones. Participants were updated between rounds by a newsletter via email and the intermediate results were also communicated via the project website. In the first survey, 39 outcomes were presented with their definitions and lay explanations. All participants were invited to rate the importance of each outcome on a 5-point Likert scale, ranging from very unimportant (1) to very important (5). They were also asked to provide reasoning behind their ratings in an optional open text field. PE were additionally asked whether they agreed with the stated definition of each outcome and to provide an alternative if they did not. Round one closed after 6 weeks, after which the results were analysed. Mean ratings for each outcome were calculated, and the three coordinating researchers (MSV, PJR, MGM) qualitatively analysed open-text answers.

In the second survey, participants were invited to consider the feedback from the first round and re-rate the outcomes. To encourage prioritization, the question was rephrased. Participants were asked to indicate their level of agreement with the statement “This outcome should be one of the ten outcomes that are always measured in research” on a 5-point Likert scale, ranging from strongly disagree (1) to strongly agree (5). Feedback from the previous round was provided by presenting the outcomes in the order ranked from the outcome that received the highest to the outcome that received the lowest mean rating. In addition, to illustrate the reasoning behind the ratings, representative quotes from stakeholder groups were presented. Following the conclusion of the second round, the results were analysed based on pre-determined consensus criteria. Outcomes were defined as ‘core’ if they had received 75% or more agreement (ratings 4 or 5) and less than 15% disagreement (ratings 1 or 2) in both stakeholder groups. Conversely, outcomes that received disagreement (ratings 1 or 2) by 50% or more participants from both stakeholder groups, were excluded from the list of potential core outcomes. Only outcomes that failed to meet the inclusion or exclusion criteria progressed to the third Delphi round.

In the third survey, the same methodology as the second round was applied to the undecided outcomes. If the agreement rating (4 or 5) differed by more than 10% between the two stakeholder groups, participants were prompted to review the reasoning provided by the participants. Based on the pre-defined criteria, outcomes without consensus after the third Delphi round were presented at the consensus meeting for a final decision.

### Phase 3–consensus meeting

Participants who had completed all three Delphi rounds were eligible to participate in the online consensus meeting and could indicate their interest at the end of the third survey. If more participants indicated their interest than could be accommodated, purposive sampling was used to ensure an equal representation of both stakeholder groups. All participants received a preparatory document in advance, provided an informed e-consent and agreed to the recording of the meeting.

A SSG meeting was convened to determine the content and structure of the meeting, as well as the definitive phrasing and definitions of all remaining (potential) core outcomes. These were informed by the feedback provided by participants in the first Delphi survey.

The consensus meeting was moderated by an independent facilitator with experience in COS development and consensus meeting moderation and delivered online via Zoom Pro (version 6.1.11). After a summary of the project and the findings to date, the outcomes that did not reach the threshold for inclusion or exclusion were discussed and voted on in clusters based on their ratings in the third Delphi round. Prior to the meeting, participants were informed that if no consensus was reached during the meeting, an executive decision would be taken by the SSG afterwards.

For each cluster, the moderator presented the outcomes and quotes to illustrate the rationales of participants in both groups. This was followed by discussion and voting. The outcomes were voted on (for inclusion or exclusion) either as a cluster using a dichotomous scale or as individual outcomes using a 5-point Likert scale. The consensus criteria were identical to those used in the Delphi process. After each electronic and anonymous round of voting, results were immediately communicated to the meeting participants. The results and the next steps were presented at the end of the meeting.

### Demographics and participation

Demographics of all participants were collected at the first survey through the LimeSurvey platform.

### Statistics

Collected data was descriptively analysed using IBM SPSS Statistics Version 28.0.0.1 (SPSS, Inc, Chicago, IL, USA). Drop-out rates between rounds were calculated.

### Role of funding source

No funding was received for this project.

## Results

### Phase 1–identification of potential core outcomes

A previous systematic review of outcome measures reported in the context of feminizing gGAS identified 2621 individual outcomes.^12^ This number was reduced to 718 unique outcomes based on similar meaning, and by removing obsolete terms and outcomes not influenced by treatment (which should thus be classified as characteristics). Analysis of transcripts from 12 interviews and three focus groups, representing data from 34 transgender and gender-diverse participants, yielded 69 outcomes. Of these, eight had not been identified by the systematic review and were added to the list. In the second step, the outcomes were sorted by level of relevance. Many outcomes were reported only once and excluded based on common sense. Complications were decided to be included separately, as they can have a significant impact and differentiate between different surgeries. Following an iterative review of all outcomes and expert consultations, 97 outcomes remained (Supplemental material S3). After categorisation, prioritisation and a structured discussion, the SSG reached a consensus on a Delphi list of 39 outcomes.

### Phase 2–Delphi process

#### Participants

**Fig. 2:**
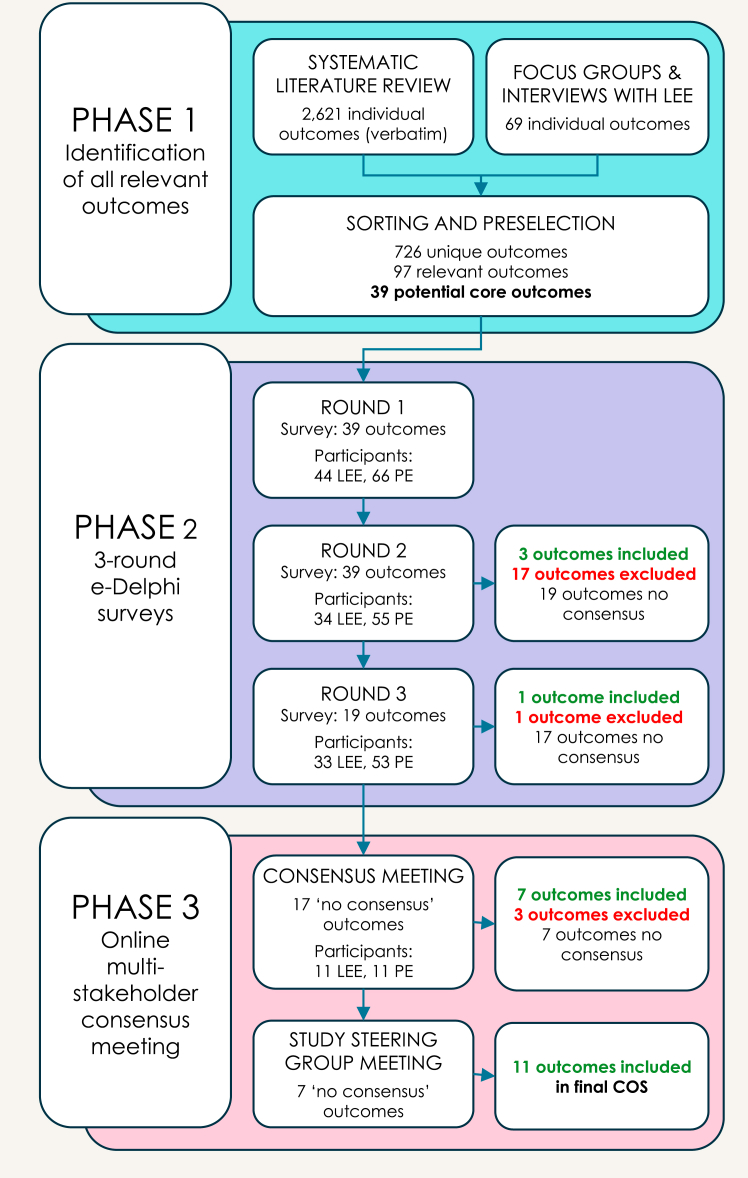
The flow chart illustrates the selection of outcomes and the number of participants at each phase of the study.

Demographics of the 86 participants who completed all three Delphi rounds are presented in Table 1. In total, 317 people registered for the Delphi study, of whom 14 were excluded as they did not meet eligibility criteria. The first survey was completed by 110 participants (44 LEE and 66 PE) from 19 countries. The second and third surveys were completed by 89 (34 LEE; 55 PE) and 86 (33 LEE; 53 PE) participants respectively. Between rounds one and two, the attrition rate was 19% (21 of 110 participants: 10 LEE and 11 PE). Only three participants (3%) dropped out after round two. An overview of the selection of outcomes and the number of participants in each phase of the study is given in Fig. 2.

**Table 1:** Demographics of participants who completed all Delphi rounds.

	LEE (n = 33)	N (%)
Gender identity	Transgender woman	28 (85%)
	Non-binary	4 (12%)
	Desister	1 (3%)
Country of residence	Argentina	1 (3%)
	Austria	1 (3%)
	Belgium	5 (15%)
	Canada	2 (6%)
	Croatia	1 (3%)
	Netherlands	6 (18%)
	Norway	3 (9%)
	UK	7 (21%)
	USA	7 (21%)
Age	18–24 years	2 (6%)
	25–34 years	10 (30%)
	35–44 years	9 (37%)
	45–54 years	5 (15%)
	55–64 years	5 (15%)
	65+ years	2 (6%)
Operations	Vaginoplasty	30 (91%)
	Vulvoplasty	4 (12%)
	Labiaplasty	4 (12%)
	Cliteroplasty	3 (9%)
Time since primary surgery	3 mo–1 yrs	14 (42%)
	1–2 yrs	4 (12%)
	3–5 yrs	8 (24%)
	6–9 yrs	5 (15%)
	10–19 yrs	1 (3%)
	>20 yrs	1 (3%)

	Prof. experts (n = 53)	N (%)
Gender identity	Transgender woman	1 (2%)
	Transgender man	1 (2%)
	Genderqueer	1 (2%)
	Cisgender woman	20 (38%)
	Cisgender man	30 (57%)
Country of residence	Argentina	2 (4%)
	Austria	2 (4%)
	Belgium	2 (4%)
	Canada	3 (6%)
	Chile	1 (2%)
	Colombia	1 (2%)
	France	1 (2%)
	Germany	1 (2%)
	Greece	1 (2%)
	Netherlands	11 (21%)
	Spain	1 (2%)
	Sweden	2 (4%)
	Taiwan	1 (2%)
	Thailand	1 (2%)
	UK	7 (13%)
	USA	16 (30%)
Age	18–24 years	0
	25–34 years	7 (13%)
	35–44 years	20 (38%)
	45–54 years	12 (23%)
	55–64 years	12 (23%)
	65+ years	2 (4%)
Occupation	Author of research	22 (42%)
	Anaesthesiologist	1 (2%)
	Endocrinologist	1 (2%)
	General/GI Surgeon	2 (4%)
	Gynaecologist	2 (4%)
	Mental health specialist	10 (19%)
	Physician assistant	2 (4%)
	Physiotherapist	2 (4%)
	Plastic surgeon	19 (36%)
	Sexologist	2 (4%)
	Urologist	13 (25%)
Duration of work experience	<2 yrs	7 (13%)
	3–5 yrs	12 (23%)
	6–9 yrs	12 (23%)
	10–19 yrs	9 (17%)
	>20 yrs	13 (25%)

LEE: Lived Exp Experts; PE: Professional Experts; UK: United Kingdom; USA: United States of America.

#### Outcome definitions

In the first survey, the agreement with the stated outcomes definitions among the PE group was high, with an average level of agreement of 91% (range 82–97%). See Supplemental material S4.1.

#### Prioritization of outcomes during the Delphi study

The first round of the Delphi study was opened in February 2024. In round one, all 39 outcomes were rated as important by both stakeholder groups, resulting in a median rating of either 4 or 5 for all outcomes, reflecting a lack of prioritization. Rephrasing of the question and Likert scale, along with additional clarification, resulted in more variation in ratings between outcomes in the second Delphi survey. In the second round, 20 of 39 outcomes met the criteria for inclusion or exclusion. Seventeen outcomes met the exclusion thresholds, and three outcomes met the inclusion thresholds. Nineteen outcomes did not reach consensus. For nine of these outcomes, the ratings differed more than 10% between stakeholder groups. Participants were alerted to these differences in the third survey, where they were asked to read the quotes before re-rating to encourage consensus between stakeholder groups. In round three, the ratings from both stakeholder groups remained largely unchanged. Only one additional outcome met the inclusion threshold, and one outcome met the exclusion threshold. The level of consensus did not increase as much as it did between rounds one and two, as ratings for 10 out of 19 outcomes still differed by more than 10% between stakeholder groups and for half of the outcomes, ratings changed 5% or less. The results of the second and third Delphi rounds are presented in Table 2.

**Table 2:** Agreement rates, results and classification after Delphi round 2 & 3.

LEE = Lived Experience Expert; PE = Professional Expert; NC = No consensus; QoL = Quality of Live; fgGAS = feminizing genital gender-affirming surgery.

Supplemental materials S4.2 and S4.3 provide details about the ranking and scores of the outcomes in each round. The quotes that were presented to participants to provide feedback are presented in Supplemental material S4.4.

The ratings of the 17 outcomes that did not reach consensus during the Delphi study were analysed and discussed by the SSG. The outcomes were grouped into four clusters (Table 2, last column).

The outcome ‘Ability to perform sexual activity as desired’, was rated very high (LEE: 75.8%, PE: 71.7%) in both stakeholder groups, but had considerable overlap with the already included outcome ‘Satisfaction with sexual function’. Therefore, the SSG proposed to merge both outcomes into one: ‘Sexual well-being’. The second cluster, labelled ‘borderline exclusion’, included seven outcomes that received low overall ratings. All but one outcome was excluded by one stakeholder group and all outcomes received less than 58% agreement by both stakeholder groups. The third cluster, ‘adverse events’, consisted of four clinically reported outcomes that met the inclusion thresholds for PE but not for LEE. The SSG decided to include the outcome ‘Need for additional surgery’ as well, because this was the highest scoring adverse event in the LEE group and rated just below the inclusion threshold by PE. The fourth cluster, labelled ‘no consensus’, included four outcomes that received divergent ratings from the two stakeholder groups. These outcomes should be discussed individually to reach consensus for either inclusion or exclusion. Based on the feedback on definitions during round one, the SSG changed the wording of two potential core outcomes, namely, ‘Effect of surgery on Quality of Life (QoL)’ to ‘Health-related QoL’ and ‘Internal (neo-vaginal) flap and or graft necrosis’ to ‘Loss of neo-vaginal tissue lining’.

### Phase 3–consensus meeting

Eleven of the 12 invited LEE and 11 of the 19 invited PE attended the meeting in September 2024. Supplemental material S5.1 provides an overview of participant characteristics.

During the 3-h meeting, eight rounds of voting were held. The voting results per group for each round are presented in Table 3. The outcomes ‘Ability to perform sexual activity as desired’ and ‘Satisfaction with sexual function’ were not merged, since the agreement rate for merging was only 63% in the LEE group. Participants from both stakeholder groups argued and voted against the separate inclusion of ‘Ability to perform sexual activity as desired’, as they felt the outcome was too vague and already covered by the outcome ‘Satisfaction with sexual function’.

**Table 3:** Results of voting at the consensus meeting.

#	Vote	Outcome	LEE vote result	PE vote result	Result
1	Merge two outcomes?	Ability to perform sexual activity as desired Satisfaction with sexual function	63% Agree (n = 7/11)	80% Agree (n = 8/10)	Not merged
2	Include this outcome in COS?	Ability to perform sexual activity as desired	40% vote 4 or 5 (n = 4/10)	40% vote 4 or 5 (n = 4/10)	Not included
3	Exclude outcomes in the cluster ‘Borderline exclusion’? (combined vote)	Neo-vaginal prolapse	60% agreement with exclusion (n = 6/10)	89% agreement with exclusion (n = 8/9)	No consensus, excluded by study steering team
		Regret type of genital gender surgery			
		External (neo-vulvar) flap and/or graft necrosis			
		Neo-meatus stenosis			
		Intra-operative rectal injury			
		Surgical result matching expectations			
		Urinary incontinence			
4	Include outcomes in the cluster ‘Adverse events’? (combined vote)	Loss of neo-vaginal tissue lining	82% agreement with inclusion (n = 9/11)	100% agreement with inclusion (n = 9/9)	Included
		Neo-vaginal stenosis			
		Need for re-intervention			
		Stricture of the neo-vaginal introitus			
		Rectovaginal fistula			
5–8	Should this outcome be included in the COS? (Separate votes)	Genital gender dysphoria	82% (n = 9/11)	78% (n = 7/9)	Included (after rephrasing)
		Satisfaction with aesthetic outcome	90% (n = 10/11)	100% (n = 11)	Included
		Genital pain during sexual activity	27% (n = 3/11)	20% (n = 2/10)	Excluded
		Ability to achieve orgasm	10% (n = 1/10)	40% (n = 4/10)	Excluded

LEE = Lived Experience Expert; PE = Professional Expert.

No decision was taken on the outcomes in the cluster ‘borderline exclusion’. All but one PE voted to exclude the outcomes, but the LEE group did not reach the predetermined threshold (63% agreement on exclusion), leaving the decision on these outcomes to the SSG after the meeting. Regarding the ‘adverse events’ outcomes, PE emphasised the value of standardized measurement of these outcomes for quality of care. The LEE group suggested that the lower agreement rates for LEE in the Delphi study were likely due to a lack of medical knowledge rather than these outcomes being considered non-critical. After discussion, participants voted to include the ‘adverse event outcomes’ (i.e., Additional surgery; Loss of neovaginal tissue lining; Neovaginal stenosis; Stricture of the neovaginal introitus; Rectovaginal fistula) (LEE: 82%. PE: 100%).

The outcomes in the fourth ‘no consensus’ cluster were discussed and voted on individually, resulting in the inclusion of ‘Satisfaction with aesthetic outcome’ and ‘Genital gender dysphoria’, provided that the phrasing of the latter outcome was changed to a more positive and inclusive term by the SSG. For an overview of the voting results, see Supplemental material S5.2.

Following the consensus meeting, the SSG decided that, since the majority of Delphi and consensus meeting participants did not consider the outcomes in the cluster ‘borderline exclusion’ critical, these outcomes should not be included. In line with the request of the consensus meeting participants, the outcome ‘Genital gender dysphoria’ was rephrased to ‘Genital gender congruence’.

### Final COS for feminizing gGAS

The final core outcome set comprises 11 outcomes as outlined in Table 4. Seven of these outcomes apply to all techniques of feminizing gGAS of which six are patient-reported outcomes, and one is a clinical outcome. Four outcomes are specific to vaginoplasty procedures and comprise adverse events associated with the creation of a vaginal canal.

**Table 4:** The final core outcome set for feminizing genital gender-affirming surgery.

Scope	#	Outcome	Type of outcome	Definition
Feminizing gGAS	1	Health related quality of life	Patient reported outcome	Evaluation of the patient’s health-related quality of life, considering biological, psychological and social functioning, after genital gender-affirming surgery.
	2	Genital gender congruence	Patient reported outcome	The degree of experienced congruence between one’s gender identity and one’s physical genitals.
	3	Satisfaction with surgical result	Patient reported outcome	Patient assessed overall satisfaction with the outcome of genital surgery, in relation to expectations, desires, and gender affirmation.
	4	Satisfaction with aesthetic outcome	Patient reported outcome	Satisfaction with the aesthetic result of the surgically created vulva.
	5	Satisfaction with neo-genital sexual function	Patient reported outcome	Satisfaction with how the surgically created genitals function sexually.
	6	Erogenous sensibility of the genitals	Patient reported outcome	Ability to experience a sexually pleasurable or arousing sensation in the genital area.
	7	Additional surgery	Clinical outcome	The need for additional surgery to deal with complications, suboptimal outcomes, perceived problems or non-achieved desired results following previous genital gender affirming surgery.
Adverse events of vaginoplasty with depth	8	Loss of neovaginal tissue lining	Adverse event	Proportion of the tissue used for lining the surgically created vaginal canal that has been lost due to ischemia infection or other causes.
	9	Neo-vaginal stenosis	Adverse event	Narrowing of the surgically created neovaginal canal resulting in decreased functional vaginal depth and/or width causing symptoms in the patient.
	10	Stricture of the neovaginal introitus	Adverse event	Narrowing of or the presence of skin bridges at the surgically constructed opening of the neovagina.
	11	Rectovaginal fistula	Adverse event	Abnormal connection between the surgically constructed neo-vagina and the rectum.

gGAS: genital gender-affirming surgery.

### Protocol deviations

Only minor adjustments were made to the study protocol, and participants were informed of these between the e-Delphi survey rounds. LimeSurvey was used as the survey platform, as our institution had terminated its license with Survalyzer. The phrasing of the questions was adapted after the first Delphi round for better understanding. Quantitative feedback on the ratings from previous rounds was provided as a ranking of outcomes based on the mean score of all participants rather than presenting medians per stakeholder group, which is more typical in Delphi procedures for COS development. Rather than asking all participants for feedback regarding the outcomes’ definitions, only PE participants were consulted. Finally, the maximum of four reminders to complete the survey specified in the protocol was extended to five to ensure a minimal drop-out rate.

## Discussion

A COS was developed through an extensive consensus process involving both professional experts in the field of gender-affirming care and people with lived experience of feminizing gGAS. A consensus was reached on the inclusion of eleven outcomes critical to measure and report in research on feminizing gGAS procedures. The COS encompasses seven outcomes applicable to all feminizing gGAS procedures and four outcomes that are specific to vaginoplasty. The adoption of this COS in future studies of feminizing gGAS will ensure that the most critical relevant outcomes are measured and reported in a standardized manner.

Selecting the precise scope of a COS is a pivotal step in its development. A deliberate choice was made to focus on feminizing genital surgery in the context of gender-affirming care, although procedures such as vaginoplasty are also employed for other indications. This specific scope facilitates the interpretation of outcomes in the context of gender-affirmation and enables comparisons between different genital affirmation procedures. In turn, this may help inform transgender and gender diverse people with decisions about genital surgery. It is also more feasible to implement a COS for a collection of procedures with the same aim and indication.

To date, research on gender-affirming surgery has been characterized by a weakness in patient-centeredness and stakeholder-inclusivity.^11^ Apart from the profound heterogeneity in reported outcomes in studies on feminizing gGAS, the most commonly reported outcomes across all studies are clinician reported, predominantly adverse events.^12^ The multi-stakeholder approach adopted in this project ensured the inclusion of the perspectives of persons with lived experience with feminizing gGAS in all stages of COS development. Contrary to the extant literature, most of the selected critical important outcomes are PROs. Of the eleven outcomes selected, six are PROs, one outcome addresses the need for additional surgery, and four are adverse outcomes, which are specific to the vaginoplasty procedure with canal creation.

The specific choice of PROs was strongly motivated by LEE participants. In articles on feminizing gGAS, ‘Attainment of orgasm’ and ‘Dyspareunia’ were among the most prevalent reported outcomes.^12^ However, during the consensus meeting, stakeholders rated ‘Ability to achieve orgasm’ and ‘Pain during sexual activity’ low. Consequently, these outcomes were excluded. Instead, consensus was reached that ‘Satisfaction with sexual function’ and ‘Erogenous sensibility of the genitals’ should be identified as core outcomes reflecting on sexual functionality. The incorporation of transgender and gender-diverse perspectives resulted in a more inclusive and less heteronormative selection of outcomes related to sexual function, thereby underscoring the value of patient-centeredness in outcome selection. It is also noteworthy that both of the selected outcomes have been documented in less than 1% of studies to date.^12^ Two further included PROs were: ‘Satisfaction with the surgical result’ and ‘Satisfaction with the aesthetic outcome’. Both stakeholder groups considered these outcomes to be complementary and relevant. The additional two PROs, ‘Health related Quality of Life (hrQoL)’ and ‘Genital gender congruence’ address more general goals of gGAS. The outcome hrQoL was selected early on, as stakeholders expressed this as the overarching goal of treatment. Although hrQoL is a commonly measured outcome in many other healthcare areas, its use in feminizing gGAS is low.^21^ Given that hrQoL depends on many factors in addition to the success or failure of surgery, its specificity for differentiating between different procedures or techniques can be questioned. However, data synthesis of this outcome can provide valuable information on the overall outcomes of gender-affirming healthcare and gGAS. The inclusion of the outcome ‘Genital gender congruence’ was particularly advocated by LEE. Many LEE argued that the ultimate goal of undergoing genital surgery is to improve this outcome. In the Delphi study, adverse events were rated higher overall by PE than LEE, specifically those with possible irreversible effects and those requiring revision surgery. These factors were also cited by PE as reasons for rating outcomes as critical in the first round. By contrast, LEE based their ratings more on the incidence of the outcome and whether (the treatment of) the adverse event would influence quality of life. During the consensus meeting, LEE indicated that their assessment of adverse events was influenced by a lack of knowledge about their incidence, impact and manageability. After discussion, consensus was reached to include the highest rated adverse events as well as the outcome ‘Need for additional surgery’. All adverse events included in the final COS are associated with the creation of a vaginal canal, emphasizing the need for reliable information on the risks associated with vaginoplasty. Regarding vaginal depth procedures, the standardized collection of adverse outcome data can better inform the choice of surgical technique and improve our evidence base for decision making.

This study has several strengths. Primarily, the inclusion of both LEE and PE in all stages of the study ensured that the results represent both stakeholder groups’ views and values equally. The high level of commitment to the project from both professional and lived experience experts was reflected in the low attrition rate of participants; 21% between rounds one and two and 3% between rounds two and three. The consensus meeting also demonstrated a high level of engagement and collaboration between the groups. The equal representation of participants from both stakeholder groups ensured an inclusive and balanced discussion where diverse perspectives were shared openly and respectfully. This commitment was further reflected in the voting results, which showed increased consensus between the groups.

We consider the provision of qualitative feedback for consensus building an additional strength of this study. The Delphi process relies on feedback from previous rounds to build consensus, typically using quantitative mean/median ratings. In this project, we provided additional qualitative feedback from both stakeholder groups in the form of quotes, aiming to provide insight into the reasoning behind the ratings from different perspectives and to encourage consensus. During the consensus meeting, participants acknowledged the added value of the qualitative data while discussing the relevance and importance of the outcome in question. While we are not aware of other COS development studies that have used qualitative feedback, we suggest that it enhanced understanding between stakeholder groups and consensus building.

This study also has several limitations. Firstly, some participation bias cannot be ruled out. The majority of participants were from Western countries. This largely reflects the origins of research into gGAS,^12^ the focus of which is COS implementation. However, the underrepresentation of Asian countries, where feminizing gGAS is also frequently performed, may have resulted in a lack of perspectives from LEE in this area. Cultural differences may affect which patient reported outcomes are deemed most important. Also, the inclusion of participants, in particular LEE participants, was likely biased by favoring digitally and language proficient people, despite offering the Delphi surveys in three languages and providing lay language explanations. It is unclear whether this affected outcome selection. Secondly, the substantial heterogeneity of outcomes was already evident from the literature review. However, the number of unique outcomes identified in Phase 1 far exceeded the number that could be included in a Delphi study, necessitating preselection. The COMET methodology does not provide guidance on the preselection of outcomes or on what constitutes a feasible number of outcomes for a Delphi study. Therefore, we consulted experts in COS development. Although the selection process was carried out carefully and in a structured way, subjective elements cannot be ruled out. To maximize transparency, we have outlined the process in detail and provided preliminary lists in this publication. Additionally, we did not explicitly request suggestions for additional outcomes in the initial Delphi round, as we believed the phase one collection process to be sufficiently rigorous. Participants were invited to provide feedback at the end of the surveys, and no one indicated that an outcome was missing. Thirdly, it can be argued that consensus building was effectively achieved in two (instead of three) Delphi rounds. In the initial Delphi round, participants were tasked with evaluating outcomes based on their level of importance. As all outcomes were deemed important, there was minimal prioritization between outcomes in the first round. However, by rephrasing the question in subsequent rounds, effective prioritization of the most critical outcomes was achieved. Also, relative high thresholds were selected for the inclusion and exclusion of outcomes. This approach ensured that outcomes were not selected too early, but it did result in a large number of outcomes being put forward for decision in the consensus meeting. Finally, the incorporation of qualitative data and the selection of illustrative quotes to present feedback represent novel elements within the Delphi methodology, and may introduce subjectivity. However, based on the discourse during the consensus meeting, we contend that these elements enhanced the consensus process.

This study has outlined the development of a COS for feminizing gGAS, representing the outcomes that should, as a minimum, always be measured and reported in clinical research on feminizing gGAS. Whilst this publication illustrates the eleven Core Outcomes of the COS, the intent is to provide a subsequent publication indicating “how” and “when” to measure these outcomes. This will further guide clinicians on how to use the COS in the clinical setting in the future. It will provide guidance on when to measure these COS outcomes and with which outcome measurement instruments. Subsequent adoption of this COS will enable comparison and syntheses of data, and enhance the evidence base in feminizing gGAS, which will in turn contribute to informed and shared decision making.

The development of this COS marks a pivotal step in two key areas: 1) standardizing outcome research and 2) ensuring stakeholder representation in outcome selection in this field. To ensure its continued applicability, the COS should be reviewed and updated periodically taking into account the limitations of the current study.^14^

## Contributors

Conceptualization: all authors contributed equally. Data curation: MSV, PJR, MA. Formal analysis: MSV, PJR, MGM. Methodology: MSV, PJR, TEP, MGM. Project administration: MSV, PJR. Resources: all authors contributed equally. Software MSV, PJR. Supervision: MGM. Translations: JB, MB, AC, MSV, PJR. Writing–original draft: MSV, MGM. Writing–review & editing: all authors contributed equally. All authors read and approved the final version of the manuscript. MSV, PJR, MA and MGM had access to and verified the underlying data.

## Data sharing statement

The data supporting this study’s findings are primarily available in the Supplemental material. Any additional data are available upon reasonable request from the corresponding author.

## Declaration of interests

Author WPB has served as a paid consultant for Karo Healthcare (Stockholm, Sweden), as an unpaid President of the WPATH (past), and as a paid Editor-in-Chief of the International Journal of Transgender Health (past). All other authors declare no conflicts of interest.
