# Supplementary material for: The core outcome set for studies on feminizing genital gender-affirming surgery: findings from the GenderCOS project
Source: eClinicalMedicine. 2025 Jul 1;85:103323. doi: 10.1016/j.eclinm.2025.103323 (PMC12308307; doi:10.1016/j.eclinm.2025.103323)
Supplement: Supplemental materials S1–S5.2 [file mmc1.pdf]

Supplementary Materials

The Core Outcome Set for Studies on Feminizing Genital Gender-Affirming Surgery – Findings from the GenderCOS Project

Table of contents

		Page
S1	COS-STAR checklist	1
S2	In- and exclusion criteria of stakeholder groups	2
S3	Longlist of 97 relevant outcomes from the systematic review and focus groups	2
S4.1	The 39 Outcomes taken to Delphi study, proposed definitions, and agreement rate with definition	4
S4.2	Order of outcomes and results of Delphi round 2	6
S4.3	Order of outcomes and results of Delphi round 3	7
S4.4	Quotes accompanying outcomes as qualitative feedback	8
S5.1	Characteristics of participants in the consensus meeting	13
S5.2	Consensus meeting voting results	14

S1 Core Outcome Set - STandards for Reporting: The COS-STAR Statement Checklist

**SECTION/TOPIC**	**ITEM No.**	**CHECKLIST ITEM**	**REPORTED ON PAGE NUMBER**
TITLE / ABSTRACT			
Title	1a	Identify in the title that the paper reports the development of a COS	1
Abstract	1b	Provide a structured summary	2
INTRODUCTION			
Background and Objectives	2a	Describe the background and explain the rationale for developing the COS.	4-5
	2b	Describe the specific objectives with reference to developing a COS.	
Scope	3a	Describe the health condition(s) and population(s) covered by the COS.	
	3b	Describe the intervention(s) covered by the COS.	
	3c	Describe the setting(s) in which the COS is to be applied.	
METHODS			
Protocol/Registry Entry	4	Indicate where the COS development protocol can be accessed, if available, and/or the study registration details.	6
Participants	5	Describe the rationale for stakeholder groups involved in the COS development process, eligibility criteria for participants from each group, and a description of how the individuals involved were identified.	6
Information Sources	6a	Describe the information sources used to identify an initial list of outcomes.	7-8
	6b	Describe how outcomes were dropped/combined, with reasons (if applicable).	
Consensus Process	7	Describe how the consensus process was undertaken.	8-10
Outcome Scoring	8	Describe how outcomes were scored and how scores were summarised.	
Consensus Definition	9a	Describe the consensus definition.	
	9b	Describe the procedure for determining how outcomes were included or excluded from consideration during the consensus process.	
Ethics and Consent	10	Provide a statement regarding the ethics and consent issues for the study.	6
RESULTS			
Protocol Deviations	11	Describe any changes from the protocol (if applicable), with reasons, and describe what impact these changes have on the results.	14
Participants	12	Present data on the number and relevant characteristics of the people involved at all stages of COS development.	11, Table 1
Outcomes	13a	List all outcomes considered at the start of the consensus process.	Supplemental materials S4.1
	13b	Describe any new outcomes introduced and any outcomes dropped, with reasons, during the consensus process.	11-13
COS	14	List the outcomes in the final COS.	14, Table 4
DISCUSSION			
Limitations	15	Discuss any limitations in the COS development process.	17-18
Conclusions	16	Provide an interpretation of the final COS in the context of other evidence, and implications for future research.	
OTHER INFORMATION			
Funding	17	Describe sources of funding/role of funders.	10, 20
Conflicts of Interest	18	Describe any conflicts of interest within the study team and how these were managed.	

From: Kirkham JJ, Gorst S, Altman DG, Blazeby JM, Clarke M, Devane D, et al. (2016) Core Outcome Set–STAndards for Reporting: The COS-STAR Statement. PLoS Med 13(10): e1002148.

S2 In- and exclusion criteria of stakeholder groups for the GenderCOS project

Stakeholder group	Inclusion criteria		Exclusion criteria
**Clinical researchers in the field of fgGAS**	Authors of at least 5 published studies into one of the surgical techniques as stated in the scope	Read and understand English, Spanish or Dutch	Researchers who have no clinical experience with individuals who have undergone fgGAS
**Surgeons and other health care professionals with experience in fgGAS**	Surgeons specialized in fgGAS (e.g. surgeons, urologists, gynaecologists, plastic surgeons)<br>or<br>Healthcare professionals who provide care around fgGAS (e.g. psychologists, endocrinologists, physiotherapist, sexologists, physician assistants)	Read and understand English, Spanish, or Dutch	Professionals in gender healthcare who have no experiences with individuals who have undergone fgGAS
**Transgender and gender diverse individuals**	Transgender women or gender diverse individuals who have undergone one of the feminizing gGAS techniques as stated in the scope at least 3 months ago and of legal age for fgGAS in the country of surgery.	Read and understand English, Spanish, or Dutch	Individuals who are unable to give informed consent, or read and understand the e-Delphi surveys

fgGAS: feminizing genital gender-affirming surgery.

S3 Longlist of 97 relevant outcomes from the systematic review and focus groups

1	Dimensions of neovagina
2	Intra-operative rectal injury
3	Post-operative bleeding
4	Urinary retention
5	Wound dehiscence
6	Infection around the operative site
7	Anastomotic leak
8	Presence of cavernosal and spongiosal remnants
9	Neovaginal stenosis
10	Meatal stenosis
11	Stricture of the neovagina introitus
12	Rectovaginal fistula
13	Diversion neovaginitis
14	Internal flap and/or graft loss
15	External flap and/or graft loss
16	Need for re-intervention
17	Post-operative time to complete recovery
18	Misdirected urinary stream
19	Vaginal malodor
20	Emotional well-being
21	Genitals matching gender identity
22	Genital gender dysphoria
23	Genital body image
24	Patient feels confident about their genitals
25	Outcome of genital surgery was as expected
26	Satisfaction with aesthetic outcome of reconstructed genitalia
27	Satisfaction with surgical result
28	Would undergo genital gender affirming surgery again
29	Sexually active with genitalia
30	Satisfaction with sexual function
31	Ability to perform sexual function as desired
32	Patient reported experience of vaginal lubrication during sexual activity
33	Genital pain during sexual activity
34	Erogenous sensibility genitals
35	Ability to achieve orgasm

36	Neovagina depth
37	Neovagina width
38	Ability to maintain vaginal capacity
39	Tactile sensitivity in reconstructed genitals
40	Haematoma
41	Neovaginal abscess
42	Pelvic or labial abscess
43	Granulation tissue
44	Readmission
45	Clinician assessment of neovaginal aesthetic outcome
46	Surgeon rated vaginoplasty results
47	Cosmetic complications
48	Requirement for cosmetic revisional genital surgery
49	Timing of commencement of sexual activity post-operatively
50	Intestinal segment viability
51	Diverting colostomy
52	Neovaginal prolapse
53	Complications
54	Fistula (anywhere in genital area)
55	Urethrovaginal fistula
56	Requirement for revisional vaginoplasty
57	Presence of painful junctional neuromas (swelling of nerve tissue) or scars
58	Duration of surgery
59	Volume of intra-operative blood loss
60	Time (from vaginoplasty) to removal of vaginal packing
61	Post-operative mobilization
62	Length of narcotic pain medication use
63	Length of hospital stay
64	Ability to urinate without significant post-void residual volume
65	Urinary incontinence
66	Pelvic floor hypertonicity (over-active pelvic floor muscles)
67	Cancer of the neovagina
68	Patient perceived feminine appearing genitoperineal region
69	Experiencing self as female
70	Satisfaction with self-image
71	Patient feels their genitals work in the way they are supposed to
72	Functional nature of neovagina as assessed by patient
73	General life satisfaction: health
74	Patient reported effect of surgery on quality of life
75	Vaginal discharge
76	Quality of vaginal mucosal discharge
77	Amount of neovaginal discharge
78	Dyspareunia (pain during or after intercourse)
79	Pelvic pain
80	Postoperative pain
81	Clitoral pain
82	Neocliteral sensation (less, normal or hypersensitive)
83	Burden of sexual reassignment surgery
84	Patient will recommend the operation they underwent to a friend
85	Overall patient satisfaction with intervention
86	Regret about type of genital surgery
87	Engagement in sexual activity
88	Engagement in sexual intercourse
89	Sexual function
90	Adequacy of neovaginal dimensions for sexual intercourse
91	Sexual arousal
92	Sexual desire
93	Satisfaction with sex life
94	Patient reported overall happiness with sexual functioning after surgery
95	Satisfaction with orgasms and sexual intercourse
96	Urethral engorgement on sexual arousal
97	Dysuria (painful micturition)

S4.1 The 39 Outcomes taken to Delphi study, proposed definitions, and agreement rate with definition

**Final feminizing gGAS COS Delphi list**			
Outcome		Proposed definition	Agreement %
1	Dimensions of neo-vagina	The depth and width of the neo-vagina measured in centimetres from the lateral sides of the introitus to the apex of the vault, as assessed by a clinician using an instrument or dilator. Time of outcome assessment must be reported.	84.8
2	Post-operative bleeding	Bleeding at the surgical site after leaving the operating theatre, as assessed clinically and/or using imaging. Outcome must be graded according to the Clavien-Dindo classification. Time of outcome assessment must be reported.	93.9
3	Wound dehiscence	Separation of the edges of surgical incisions or wounds, primarily closed during surgery, as assessed by a clinician. Outcome must be graded according to the Clavien-Dindo classification. Time of outcome assessment must be reported.	90.9
4	Wound infection	Clinical signs of surgical site infection, as assessed by a clinician with or without a change in management. Outcome must be graded according to the Clavien-Dindo classification. Time of outcome assessment must be reported.	81.8
5	Granulation tissue	The presence of excess granulation tissue, in the genital area, as assessed by a clinician. Outcome must be graded according to the Clavien-Dindo classification. Time of outcome assessment must be reported.	89.4
6	Internal (neo-vaginal) flap and/or graft necrosis	Proportion (as an estimated percentage) of the flap or graft, used to surgically construct the vaginal vault, that has died due to ischaemia, as assessed by a clinician. Outcome must be graded according to the Clavien-Dindo classification. Time of outcome assessment must be reported.	86.9
7	External (neo-vulvar) flap and/or graft necrosis	Proportion (as an estimated percentage) of the skin flap, used to surgically construct the external vulva and clitoris, that has died due to ischaemia, as assessed by a clinician. Outcome must be graded according to the Clavien-Dindo classification. Time of outcome assessment must be reported.	93.9
8	Intestinal anastomotic leak	Failure or breakdown of the intestinal anastomosis causing leakage of intestinal contents into the abdominal cavity, as assessed clinically and/or using imaging. Outcome must be graded according to the Clavien-Dindo classification. Time of outcome assessment must be reported.	84.8
9	Intra-operative rectal injury	Surgical damage to the rectum, adjacent to the neo-vaginal space. Ranging from abrasions, tears, to complete perforation into the rectal lumen, as assessed clinically. Outcome must be graded according to the Clavien-Dindo classification.	95.5
10	Rectovaginal fistula	An abnormal connection between the surgically constructed vagina and the rectum, assessed clinically and/or using imaging. Outcome must be graded according to the Clavien-Dindo classification. Time of outcome assessment must be reported.	92.4
11	Diversion neo-vaginitis	Chronic inflammation of the mucosa of the intestinal segment used to form the neo-vaginal canal, as assessed clinically and/or by neo-vaginal biopsy. Outcome must be graded according to the Clavien-Dindo classification. Time of outcome assessment must be reported.	89.4
12	Neo-vaginal prolapse	Partial or complete protrusion of the neo-vaginal lining beyond the introitus, as assessed by a clinician. Outcome must be graded according to the Clavien-Dindo classification. Time of outcome assessment must be reported.	97.0
13	Deviated urinary stream	The experience that the urine stream is not central (off-centre) or too wide (spraying), as reported by the patient. Outcome must be graded according to the Clavien-Dindo classification. Time of outcome assessment must be reported.	89.4
14	Urinary incontinence	Unintentional passing of urine. assessed as stress-, urge- or mixed incontinence by a clinician. Outcome must be graded according to the Clavien-Dindo classification. Time of outcome assessment must be reported.	95.5
15	Urinary retention	Retention of urine in the bladder due to inability to urinate or to completely empty the bladder, as assessed by a clinician using catheter drainage and/or imaging. Outcome must be graded according to the Clavien-Dindo classification. Time of outcome assessment must be reported.	86.4
16	Neo-meatus stenosis	Narrowing of the surgically constructed urethral opening, resulting in lower urinary tract symptoms, as assessed by a clinician. Outcome must be graded according to the Clavien-Dindo classification. Time of outcome assessment must be reported.	89.4
17	Presence of surplus cavernosal and spongiosal remnants	Presence of redundant corpora cavernosa- and/or spongiosa tissue after shortening of the urethra and penectomy, that leads to patient discomfort, as assessed by a clinician. Outcome must be graded according to the Clavien-Dindo classification. Time of outcome assessment must be reported.	93.9

18	Neo-vaginal stenosis	Narrowing of the vaginal canal, resulting in a decrease of functional vaginal depth and/or width, leading to complaints, as assessed by a clinician. Outcome must be graded according to the Clavien-Dindo classification. Time of outcome assessment must be reported.	97.0
19	Stricture of the neo-vaginal introitus	Narrowing of the surgically constructed opening of the neo-vagina, as assessed by a clinician. Outcome must be graded according to the Clavien-Dindo classification. Time of outcome assessment must be reported.	93.9
20	Need for re-intervention	Medical or surgical intervention needed to deal with a complication or problem related to the previous genital gender affirming surgery. Time of re-intervention assessment must be reported.	89.4
21	Readmission	Unplanned readmission needed after discharge, due to complications or problems related to the previous genital gender affirming surgery. Time of outcome assessment must be reported.	89.4
22	Time until complete recovery	Time taken to fully heal and return to their normal level of activity and pre-operative physical state, as reported by the patient. Time of outcome assessment must be reported.	92.4
23	Requirement for cosmetic revision surgery of the genitals	Need for additional genital surgery to improve the aesthetic results of the previous genital gender affirming surgery, as assessed by a clinician and reported by the patient. Outcome must be graded according to the Clavien-Dindo classification. Time of outcome assessment must be reported.	90.9
24	Satisfaction with aesthetic outcome	Satisfaction with the aesthetic result of the surgically created vulva, as reported by the patient. Time of outcome assessment must be reported.	93.9
25	Satisfaction with surgical results	Satisfaction with the surgical result, as reported by the patient. Time of outcome assessment must be reported.	87.9
26	Surgical result matching expectations	Extent to which the surgical result match the pre-surgical expectations, as reported by the patient. Time of outcome assessment must be reported.	90.9
27	Effect of surgery on quality of life	Quality of life, as reported by the patient, pre- and post-genital gender affirming surgery. Time of outcome assessment must be reported.	97.0
28	Genitals matching gender identity	Extent to which the genitals match the gender identity, as reported by the patient. Time of outcome assessment must be reported.	93.9
29	Genital gender dysphoria	Distress and/or discomfort related to the incongruence between the gender identity and the physical genitals, as reported by the patient. Time of outcome assessment must be reported.	81.8
30	Feeling confident about genitals	Feeling of confidence about their genitals, as reported by the patient. Time of outcome assessment must be reported.	93.9
31	Willingness to undergo genital gender surgery again	Whether, with the benefit of hindsight, the same surgical treatment would be chosen again, as reported by the patient. Time of outcome assessment must be reported.	97.0
32	Regret type of genital gender surgery	Regret about the type of previous genital gender affirming surgery, as reported by the patient. Time of outcome assessment must be reported.	93.9
33	Sexually active with genitals	Use or involvement of genitals, while engaging in sexual activity, as reported by the patient. Time of outcome assessment must be reported.	87.9
34	Satisfaction with sexual function	Satisfaction with the way the genitals function in relation to the sexual needs, as reported by the patient. Time of outcome assessment must be reported.	87.9
35	Ability to perform sexual function as desired	Ability to function sexually with genitals and desired, as reported by the patient. Time of outcome assessment must be reported.	92.4
36	Erogenous sensibility of the genitals	Ability to perceive erogenous sensation in the genital area, as reported by the patient. Time of outcome assessment must be reported.	93.9
37	Ability to achieve orgasm	Ability to experience a sexual climax, as reported by the patient. Time of outcome assessment must be reported.	93.9
38	Genital pain during sexual activity	Pain in the genital area during engagement in sexual activity, as reported by the patient. Time of outcome assessment must be reported.	92.4
39	Vaginal malodour	Unpleasant or foul smell that originates from the surgically constructed neo-vagina, as reported by the patient and/or assessed by a clinician. Outcome must be graded according to the Clavien-Dindo classification. Time of outcome assessment must be reported.	86.4

S4.2 Order of outcomes and results of Delphi round 2

		Percentage of responses rated 4&5 on Likert scale		
**Rank**	**Outcome**	**Delphi R2 LEE (n=34)**	**Delphi R2 PE (n=55)**	**RESULT R2**
1	Effect of surgery on QoL	84.8	78.2	IN
2	Rectovaginal fistula	51.5	65.5	NC
3	Intra-operative rectal injury	45.5	56.4	NC
4	Internal (neo-vaginal) flap and or graft necrosis	72.7	63.6	NC
5	Neo-vaginal stenosis	72.7	80.0	NC
6	Satisfaction with sexual function	78.8	80.0	IN
7	Erogenous genital sensibility	78.8	63.6	NC
8	Intestinal anastomotic leak	39.4	45.5	OUT
9	Stricture of neovaginal introitus	57.6	56.4	NC
10	Satisfaction with surgical result	78.8	80.0	IN
11	Genital gender dysphoria	51.5	67.3	NC
12	External (neo-vulvar) flap and/or graft necrosis	48.5	58.2	NC
13	Need for re-intervention	57.6	69.1	NC
14	Regret type of genital gender surgery	51.5	54.5	NC
15	Genital pain during sexual activity	54.5	38.2	NC
16	Ability to achieve orgasm	60.6	67.3	NC
17	Vaginal dimensions	36.4	47.3	OUT
18	Satisfaction with aesthetic outcome	63.6	72.7	NC
19	Ability to perform sexual activity as desired	69.7	74.5	NC
20	Genitals matching gender identity	45.5	49.1	OUT
21	Neo-vaginal prolapse	54.5	50.9	NC
22	Urinary incontinence	57.6	52.7	NC
23	Neo-meatus stenosis	30.3	50.9	NC
24	Readmission	39.4	41.8	OUT
25	Wound dehiscence	39.4	38.2	OUT
26	Presence of surplus cavernosal and/or spongiosal remnants	51.5	40.0	NC
27	Surgical result matching expectations	57.6	50.9	NC
28	Post-operative bleeding	24.2	36.4	OUT
29	Willingness to undergo fgGAS again	42.4	36.4	OUT
30	Wound infection	24.2	34.5	OUT
31	Feeling confident about genitals	45.5	40.0	OUT
32	Urinary retention	33.3	40.0	OUT
33	Diversion neovaginitis	33.3	32.7	OUT
34	Sexually active with genitals	27.3	41.8	OUT
35	Vaginal malodour	36.4	23.6	OUT
36	Granulation tissue	33.3	25.5	OUT
37	Deviated urinary stream	21.2	29.1	OUT
38	Time until complete recovery	48.5	29.1	OUT
39	Requirement for revision surgery of genitals	42.4	47.3	OUT

NC: No Consensus, IN: Included in COS, OUT: Excluded from COS, LEE: Lived Experience Experts, PE: Professional Experts

S4.3 Order of outcomes and results of Delphi round 3

		Percentage of responses rated 4&5 on Likert scale		
**Rank**	**Outcome**	**Delphi R3 LEE (n=33)**	**Delphi R3 PE (n=53)**	**Result R3**
1	Neo-vaginal stenosis	63.6	75.5	NC
2	Ability to perform sexual activity as desired	75.8	71.7	NC
3	Erogenous sensibility of the genitals	84.8	75.5	IN
4	Internal (neo-vaginal) flap and/or graft necrosis	66.7	71.7	NC
5	Genital gender dysphoria	69.7	50.9	NC
6	Need for re-intervention	60.6	71.7	NC
7	Ability to achieve orgasm	54.5	62.3	NC
8	Satisfaction with aesthetic outcome	60.6	69.8	NC
9	Rectovaginal fistula	51.5	79.2	NC
10	Stricture of the neovaginal introitus	54.5	75.5	NC
11	Urinary incontinence	57.6	54.7	NC
12	External (neo-vulvar) flap and/or graft necrosis	48.5	52.8	NC
13	Surgical result matching expectations	54.5	37.7	NC
14	Intra-operative Rectal injury	42.4	56.6	NC
15	Neo-vaginal prolapse	51.5	43.4	NC
16	Regret type of genital gender surgery	51.5	41.5	NC
17	Genital pain during sexual activity	60.6	34.0	NC
18	Presence of surplus cavernosal and spongiosal remnants	48.5	45.3	OUT
19	Neo-meatus stenosis	42.4	56.6	NC

NC: No Consensus, IN: Included in COS, OUT: Excluded from COS, LEE: Lived Experience Experts, PE: Professional Experts

S4.4 Quotes accompanying outcomes as qualitative feedback

No	Outcome	Reasoning given by participants
1	Effect of surgery on QoL	- "QoL is influenced by many things, is it really the surgery that changed the QoL? QoL can vary from day to day."<br>- "This is, in essence, the "point" of the surgery - to improve the quality of life. Even if someone didn't get the expected depth or appearance, quality of life can be significantly improved."<br>- "This data would likely reinforce the importance of gender-affirming surgical options for trans patients. To prove that surgery is useful."<br>- "Complications of surgery can lower QoL but better mental wellbeing and less dysphoria can improve QoL, so maybe it is not specific enough and we better measure things that affect QoL."
2	Rectovaginal fistula	- "Important because it could lead to additional surgery and discomfort. Associated with poor outcome, patient satisfaction and Quality of Life."<br>- "This data point is already regularly collected. More and updated data cannot hurt, but will likely not bring revelations to light."<br>- "It is important, but I don´t think it is a frequent issue, so therefore not a core outcome."<br>- "A disastrous complication both for the patient and for the surgeon and can compromise functionality, physical outcome and has a significant impact on mental health."
3	Intra-operative Rectal injury	- "I would rate this higher if it was more common."<br>- "Very important because it can be life threatening and lead to large life changes like having an stoma while it heals. It can prolong a person's recovery and worsen mental health."<br>- "It is important but if detected in time and repaired, it may not have consequences. More important is, how many proceed to develop a fistula?<br>- "Important bench-mark to allow comparisons between surgeons and centers."
4	Internal (neo-vaginal) flap and/or graft necrosis	- "This complication impacts dimensions and function and ability to have penetrative sex. Predictor for reintervention and secondary vaginoplasty."<br>- "It is difficult to measure." It is better to measure the consequences; stenosis, lack of depth, distress etc."<br>- "Necrosis can cause long term loss compared to something easily fixable. Can be traumatic. A functional vaginal vault is the aim of the surgery."<br>- "Complications like this show how good the technique is. It causes serious consequence for those affected."
5	Neo-vaginal stenosis	- "A relevant outcome but primarily influenced by adherence to dilatation protocol and not the operation." It affects function and satisfaction but is not dangerous."<br>- "I lost 3 inches of depth even with regular dilation." This is very common and we need to understand why, how to prevent and correct." It prevents sexual enjoyment on both parties."<br>- "While this might depend on the patient's adherence to the dilation schedule, I think it is important to statistically measure how different techniques result in different depth."
6	Satisfaction with sexual function	- "Essential outcomes for most people's happiness and QoL. Satisfaction is more important than activity or whether and how they use it."<br>- "satisfaction relates more to a person's circumstances, psychosexual function and post history than the actual surgery<br>- "It's important but now too generic. probably needs to be subsumed, specified into a smaller group of sexual questions."<br>- "Among those who wish to be sexually active, this would be an important metric to understand the mix of psychological and physiological implications that surgery can have on a patient's sex life."
7	Erogenous sensitivity of the genitals	- "Important and relevant for patient satisfaction, very often requested information during pre-operative counselling<br>- "Is also dependent on other factors than the surgery. For some, orgasm may be more important."<br>- "Is an important quality of life issue and for sex life, but I prefer satisfaction with sexual function as you can measure this with a validated questionnaire<br>- "Very important for pleasure, intimacy and to feel yourself (literally and figuratively). Can show differences between techniques."
8	Intestinal anastomotic leak	- "Important but not frequent complication of colovaginoplasty with significant impact on well-being and time till recovery<br>- "Fundamental due to being a serious, possible life-threatening complication requiring immediate attention and return to theatre."<br>- "This outcome only applies to colonic vaginoplasty, which is not the standard operation. I would not consider it to be a core outcome for all vaginoplasty cases."<br>- "When I had my surgery, I wanted to know the chance of having to have a stoma. The surgeon could not tell me a quantifiable answer to what the chance was, and I wonder if it's because the data wasn't there."

9	Stricture of the neovaginal introitus	- “Stricture of the introitus may be associated with bleeding, tissue necrosis and surgical technique. Will require surgical revision if impacting patient quality of life and/or psychosexual functioning<br>- “To know how often and how long dilatation is needed to prevent the stricture would be useful for the patients to know as dilatation is time consuming.”<br>- “It can lead to loss of depth and width in the rest of the canal. Patient dependent whether this is experienced as a problem. Relevant, but not core.”<br>- “Definitely important, although surgery and dilation can help resolve it; a stricture can have a high impact on the persons' sex life and satisfaction.”
10	Satisfaction with surgical result	- “A key subjective outcome relevant to overall surgical success, Self-reported satisfaction is the best way to capture whatever happens to be important to each individual.”<br>- “The outcome is too vague, too broad. It is subjective. Not useful for comparisons.”<br>- “In my opinion as the surgery is very medically necessary for the individuals who undergo it, it's not as much about satisfaction with surgical results as it is about safety.”<br>- “I think it's important to know whether folks believe that having their gender affirming surgery was in fact; affirming.”
11	Genital gender dysphoria	- “This should be assessed before any planned surgery. It is a reason not an outcome. Genital surgery is only indicated to relieve Genital Gender Dysphoria.”<br>- “Even though it is already shown that gender affirming surgery decreases gender dysphoria, a standardized measurement would contribute to the evidence.”<br>- “I had dysphoria about my genitals but I kind of denied how bad it was. I thought before surgery that it would probably improve but it improved a lot. I feel so much better in my skin now.”<br>- “Too much weight is already given to the idea of "gender dysphoria" as a medical diagnosis. It can be a problematic label; we should be focusing on the broader scope of the human gender dynamic.”
12	External (neo-vulvar) flap and/or graft necrosis	- Need to measure the incidence of external necrosis since it can affect vulvar appearance, sexual function, clitoral sensitivity, urinary function, patient satisfaction and need for revisions.”<br>- “You should expect revisions after operation.” It's less important than everything else listed.”<br>- “The appearance is important off course but function and sensibility is more important.”<br>- “Having a within-normal-variance natural appearance is important for your self-image and mental health in terms of gender congruence and dysphoria.”
13	Need for re-intervention	- Re-intervention says something about the extra burden and the impacted quality of life and possible sexual and social burden.”<br>- “I think this will be captured in the other outcomes measured, because you have to grade complications under Clavien-Dindo.”<br>- “Subjective, because whether a patient opts for revision (for instance cosmetic) can be subjective and there is a difference between surgeons in when they prefer to re-operate, when faced with similar cases.”<br>- “Very important, also important to measure how serious and time sensitive it is. It makes a huge difference if there is some slight discomfort when aroused or life-threatening infection.”
14	Regret type of genital gender surgery	- “It may be affected by other factors than type of procedure, like some complication, prolonged return to activities or lack of care and not really by the technique performed.”<br>- “Obviously, we will need to be careful on how this is reported and regret over type of surgery is very different from the regret of transitioning entirely, this outcome could be misused.”<br>- “Feels like the reason for the regret is what is most important, and that is likely captured by other items.”<br>- “Patient would have to have undergone at least two different methods to be able to judge.”
15	Genital pain during sexual activity	- “Not necessarily dependent on surgery. Usually time-limited and counseling plays a big role (use of lubrication and pelvic floor relaxation).”<br>- “If the pain is high enough, the person may not be able to participate in sexual activities which would decrease quality of life. Attention and treatment of this is important.”<br>- “I would rate it slightly lower than orgasm and overall satisfaction with sexual function, as there are more elements to sexual pain and more treatments available to remedy genital pain during sexual activity.”
16	Ability to achieve Orgasm	- “Achieving an orgasm depends on so many things. Many patients have had few or no sexual encounters and/or an orgasm prior to surgery due to dysphoria thus post op inability to orgasm does not necessarily resemble anatomical success of operation.”<br>- “Erogenous sensibility is a more salient question in my opinion.”<br>- “Having an orgasm is nice but I wouldn't lay awake about it, if I didn't have one.”<br>- “This is part of sexual function and is important for quality of life.”

17	Vaginal dimensions	- "Adequate dimensions are important for comfort and pain-free penetrative sex. It influences self-image and sexual satisfaction."<br>- "Size is 'not everything' - function is more important. Vaginal depth is overemphasized."<br>- "Immediate post-operative depth reflects technical success but long-term dimensions are determined by dilatation and possible complications. It should be researched how much depth is lost after operation."<br>- "Width and depth of the vaginal canal is very important to me as I hope to have penetrative sex. I find this the most gender affirming form of intimacy."
18	Satisfaction with aesthetic outcome	- "Already a frequently studied topic, however a standardized measure would be great for comparison reasons of the various techniques so techniques could be adapted in the future."<br>- "This, as well as function, greatly impacts reduction in dysphoria and improves mental health."<br>- "This varies with time and is largely dependent on when it is measured and how long it takes for a person to adjust psychologically. Not a useful measure."
19	Ability to perform sexual activity as desired	- "This outcome might be an important reason to perform a genital surgery. It could help to identify reasons why the sexual function is not fulfilled."<br>- "Among those who wish to be sexually active, this would be an important metric to understand the mix of psychological and physiological implications that surgery can have on a patient's sex life."<br>- "I find this nuance confusing (assessing ability but not satisfaction) and cannot feel strongly about its inclusion or exclusion in the study."<br>- "I feel like this is just redundant with 'Satisfaction with sexual function', and doesn't give much further insight. I have bias as a lesbian here maybe, straight persons might be concerned more here?
20	Genitals matching gender identity	- "This is complicated as we get into the question of what does a 'non-binary' genitalia look like. I think we should focus on how the surgery affects gender dysphoria."<br>- "Genital congruence is associated with good psychosexual functioning and improves QoL."<br>- "To someone who chose to undergo the surgery to alleviate their genital dysphoria this would be very important and to someone who chose to do so more for comfort and sexual reasons less so."<br>- "As a non-binary person who got feminizing genital surgery, this can be a complicated question. Trans women or NB persons who do not wish to have surgery, have as much '*matching genitals to their gender identity'* as I do."
21	Neo-vaginal prolapse	- "Neo-vaginal prolapse may be associated with poor surgical technique. Important for comparing techniques, surgeons and centers."<br>- "This is a serious complication with potentially significant physical consequences, requiring surgery and therefore I rate this more important than the other (generally) minor surgical complications, which can be remedied easier."<br>- "This affects function and aesthetics and would defeat the purpose (a functional vagina) of having the surgery if not fixed."<br>- "Judging from aftercare leaflets this looks temporary as it is manageable, compared to other complications. It can also happen in Cis women."
22	Urinary incontinence	- "Relevant and annoying outcome, and common in the early stages after surgery. The degree is important but can also be influenced by many other factors."<br>- "Severe incontinence is not a very common complication after vaginoplasty, to my knowledge. I think Core Outcomes should be the outcomes that say something about the operation."<br>- "Affects quality of life and potentially has an impact on patient's sex life and social activities."<br>- "Less important because it can be prevented and does not always require operation, just pelvic physiotherapy."
23	Neo-meatus stenosis	- "Uncommon and only important when a person has symptoms. Can lead to retention of urine which can cause bladder and renal problems. However, easy to treat."<br>- "Short and long term consequence that affect the patient's quality of life."<br>- "Anything involving really struggling to pee is important because we do it so much."<br>- "Bothersome when it happens but I would hope that this complication could be resolved in a relatively minor surgery and would not be serious."
24	Readmission	- "It is a very unspecific outcome as it is not clear what the reason for readmission was. Already included in other complication outcomes if you grade it. Not all readmissions are of the same severity."<br>- "I think this could be useful but also misleading in comparing outcomes as you might be readmitted at one place for a reason that another provider would treat in an outpatient setup."<br>- "relevant, because it is a burden to the patient, but could also be due to other reasons, so not as specific for the technique or surgery as some of the other relevant outcomes."<br>- "One of the most important quality metrics is 90-day readmission rate. Data can help manage expectations."
25	Wound dehiscence	- "Wound dehiscence impacts healing time, pain and sometimes aesthetics. This way it is troublesome for the patient and impacts the recovery experience."<br>- "It is very common and seldom affects long-term results or overall satisfaction."<br>- "This is not specific for the type of genital operation, it can happen in any surgery."

26	Presence of surplus cavernosal and spongiosal remnants	- "It limits vaginal functionality (impairs penetration) and vulvar aesthetics with arousal. But how to measure? It is subjective."<br>- "I think of this potentially negative effects on genital appearance, sexual function and body dysphoria. Can be painful."<br>- "Reflects the adequacy of extirpation at the time of index vaginoplasty."<br>- "Everybody I know who had this problem, ultimately had it corrected because you want a functioning vagina after all. I do feel I did not get enough information pre-operative about this."
27	Surgical result matching expectations	- "More a reflection of pre-operative education, counseling and preparation than the actual result. Are we always clearly understanding the patients expectations?"<br>- "If the genital surgery fails to meet these goals and expectations, this negatively impacts a person's psychosexual functioning."<br>- "Results can be used to modify/improve patient education which is highly relevant to overall surgical success."<br>- "A lot of trans people I've known, even myself, build up a certain outcome. Often that isn't how it turns out. Caregivers can be important perception shapers."
28	Post-op bleeding	- "Bleeding can lead to hematoma which can have aesthetic complications as well as affecting patient satisfaction."<br>- "This is a common outcome indicator for surgical studies and can impact a patient's experience and increases the risk of bad outcome."<br>- "Even if assessed, we cannot change it and there are other outcomes that are more relevant for future research."<br>- "From my experience this is something that usually happens right after the main surgery and although it can extent hospital stay and recovery time, it doesn't cause long term problems."
29	Willingness to undergo gGAS again	- "Good measure of satisfaction with surgical outcome, as it measures the whole surgical experience."<br>- "I don't think this outcome would be helpful." Even when you're not fond of the surgery, the surgical method, the complications, the team etc; one would still undergo the same surgery if only for the simple reason that they could not exist with the alternative."<br>- "These types of questions are difficult, especially when answering a hypothetical question that is unlikely to be required."
30	Wound infection	- "While it is important to collect data on wound infection, there is already significant data available on rates of infection post-surgery for vaginoplasty. More and updated data will not bring revelatory information to the field."<br>- "If risks are higher than similar medically necessary surgery, the procedure should be questioned. Has a high impact on experience and use of healthcare."<br>- "Wound infections are rare but can determine the outcome, sometimes impairs healing, creates scars, worsen aesthetics, might require hospitalization."
31	Feeling confident about genitals	- "It does not determine your identity; it can only be related to psychological moments or situations."<br>- "This was the driving factor for me to endure surgery and the pre-op/post-op activities required to be able to have the surgery e.g. electrolysis, dilation, etc."<br>- "A person's confidence is important to their overall quality of life but in most interactions you have your pants on and people don't see your genitals so less important."<br>- "I'm not sure that this isn't covered already by several of the other outcomes. It may not be affected by surgery alone: how many cis women are confident about their genitalia?
32	Urinary retention	- "Important but rarely a long-term problem, relatively easy to support with intermittent catheterization, which can have an impact on the quality of life."<br>- "Impacts the patient experience but is usually transient. In my experience, this does not translate into patients with long term lower urinary tract symptoms or meatal stenosis."<br>- "I don't know how much the operation influences this versus pre-existent urinary problems and pelvic floor problems. Unspecific and therefore not core in my opinion."<br>- "I know someone who had to use a catheter for several months and her life was seriously impacted."
33	Diversion neovaginitis	- "It compromises vaginal functionality and often limits penetration. Important for decision making as I would not want to deal with the odor or other symptoms."<br>- "Complication that has been little investigated so far and therefore important for future studies. Risk should always be communicated to the patient because it can seriously impact quality of life."<br>- "This is a bothersome long-term chronic complication but I don't think it is influenced by technique or surgeon. Just bad luck and a consequence of using colon. Therefore not useful for comparison."<br>- "I think it is too specific for a COS, only a small percentage undergo bowel vaginoplasty. All bowel vagina's have a degree of vaginitis."
34	Sexually active with genitals	- "Should align with patient sexual goals. Lack of use is not a poor outcome if patient did not have goal of sexual penetration/partner/etc."<br>- "Sex is important for a lot of persons and genital operations can influence sexual functioning positively or negatively."<br>- "Patients have different wishes for their genital. Doesn't say much about the function, satisfaction or the quality of the surgery."

35	Vaginal malodour	- “It is not always an index of the type of surgery, may have multi factorial reasons. If odour is expected to be part of the outcome, It would be a reason for me to choose another procedure.”<br>- “Everybody has a different odour, this is normal.”<br>- “Embarrassing, would not engage in sexual activity and fear that it would be detected by others. Would impact my sex life and well-being.”<br>- “A sign that something is wrong, but can often be treated.”
36	Granulation tissue	- “This complicated is not specific for the type of surgery, it belongs to general surgery risks.”<br>- “Very common, it is part of the natural healing process and is often easily resolved without lasting effects.”<br>- “It's a nuisance but does not have a huge impact on wellbeing compared to other outcomes.”<br>- “It can affect aesthetics, cause pain, extend recovery and increase needed visits to the clinic/hospital.”
37	Deviated urinary stream	- “There is a degree to which this is about learning to urinate and the subsiding of swelling. Where this is problematic beyond that level; it does have a negative impact on wellbeing.”<br>- “Though important, I'm not sure this is CORE. In the first 3 months spraying is expected. Also common in cis females.”<br>- “It's nice not to be messy, but at the end of the day it doesn't reduce function and less unimportant than other complications.”<br>- “More annoying than really bothersome. I have this a little bit, but considering how many surgeries I have had, I have no complaints.”
38	Time until complete recovery	- “This probably varies greatly from person to person, but an average would still be important to be able to estimate when you can carry out certain activities without risk and plan with work etc.”<br>- “Very subjective measure, depends on the activity prior to surgery and is affected by so many things beside surgery.”<br>- “The recovery period is very unpleasant but in the end, a month or two is short compared to the average human lifespan this would be less of a concern.”<br>- “Very important to measure as it gives an important window into what is normal recovery time and also the outliers that require a lot longer to recuperate. Could be different between techniques.”
39	Requirement for revision surgery of genitals	- “Not sure this is a relevant outcome since some surgeons may be more lenient or open to cosmetic revisions than others and in some countries, it is not funded.”<br>- “I would rate this as less important can be due to unrealistic views of what genitalia should look like.”<br>- “There is an overlap with the ‘Satisfaction with aesthetic outcome’. I prefer this outcome measure as it reflects shared-decision making between patient and doctor.”<br>- “Cosmetic outcome is subjective, hard to quantify and not as important as functional outcome in my opinion. Measures both the patient and professional point of view and that lowers the quality of the outcome.”

S5.1 Characteristics of participants in the consensus meeting

#	Present	Gender identity	Role	Time since first fgGAS	Country
1	yes	Transgender woman	LEE	10-19 yrs	United States of America
2	yes	Transgender woman	LEE	3 mo- 1 yrs	United States of America
3	yes	Transgender woman	LEE	1-2 yrs	Austria
4	yes	Transgender woman	LEE	3 mo- 1yrs	Belgium
5	yes	Transgender woman	LEE	3 mo- 1 yrs	United Kingdom
6	no	Non-binary	LEE	3mo- 1 yrs	United States of America
7	yes	Transgender woman	LEE	3mo- 1 yrs	The Netherlands
8	yes	Transgender woman	LEE	3mo- 1 yrs	Norway
9	yes	Transgender woman	LEE	3-5 yrs	The Netherlands
10	yes	Transgender woman	LEE	3-5 yrs	Canada
11	yes	Transgender woman	LEE	3-5 yrs	The Netherlands
12	yes	Transgender woman	LEE	3 mo-1yrs	Belgium
				**Work experience**	
13	yes	Cisgender man	Author* & Urologist	6-9 yr	Germany
14	yes	Cisgender woman	Author & Urologist	6-9 yr	United States of America
15	no	Cisgender man	Plastic surgeon	3-5 yr	United Kingdom
16	no	Cisgender man	Author & Urologist	20+ yrs	United States of America
17	no	Cisgender man	Author & Plastic surgeon	20+ yrs	United States of America
18	no	Cisgender man	Author & Psychiatrist	20+ yrs	United Kingdom
19	yes	Cisgender woman	Psychologist	6-9 yrs	United Kingdom
20	yes	Cisgender man	Author & Plastic surgeon	20 yrs	Sweden
21	yes	Cisgender man	Author & Plastic surgeon	20+ yrs	Greece
22	yes	Cisgender man	Urologist	6-9 yrs	United States of America
23	yes	Cisgender man	Physiotherapist	3-5 yrs	Taiwan
24	yes	Cisgender woman	Plastic surgeon	6-9 yrs	United States of America
25	no	Cisgender man	Author & General/plastic surgeon	6-9 yrs	Sweden
26	no	Cisgender woman	Psychologist	10-19 yrs	United States of America
27	yes	Cisgender woman	Psychologist	<2 yrs	United States of America
28	no	Cisgender woman	Plastic surgeon	3-5 yrs	The Netherlands
29	no	Cisgender man	Author & Plastic surgeon	6-9 yrs	The Netherlands
30	yes	Cisgender woman	Gynaecologist	3-5 yrs	United States of America
31	yes	Cisgender man	Author & Psychiatrist/Sexologist	6-9 yrs	The Netherlands

* 'Author' is defined as having published at least five papers in gender care. LEE: Lived Experience Experts PE: Professional Experts. Yrs: Years

S5.2 Consensus meeting voting results

No.	Question	Vote type*	Vote result % Agree (n)		Result
			LEE	PE	
**1**	Do you agree that the included outcome “Satisfaction with sexual function” and the non-included outcome “Ability to perform sexual activity as desired” should be merged into one outcome called “Sexual well-being”?	Binary	63% Agree (n=7/11)	80% Agree (n=8/10)	NOT MERGED
**2**	How much do you disagree or agree that the outcome “Ability to perform sexual activity as desired” should be **included** in the COS?	Likert Scale	40% (n=4/10)	40% (n=4/10)	OUT
**3**	The outcomes in the category borderline exclusion should **not be included** in the Core Outcome Set	Binary	60% (n=6/10)	89% (n=8/9)	UNDECIDED
**4**	The outcomes in the category ‘’Adverse events” should be **included** in the Core Outcome Set	Binary	82% (n=9/11)	100% (n=9/9)	IN
**5**	How much do you disagree or agree that the outcome ‘’Genital gender dysphoria‘’ should be **included** in the Core Outcome Set?	Likert Scale	82% (n=9/11)	78% (n=7/9)	IN
**6**	How much do you disagree or agree that the outcome ‘Satisfaction with aesthetic outcome‘’ should be **included** in the Core Outcome Set?	Likert Scale	90% (n=10/11)	100% (n=11)	IN
**7**	How much do you disagree or agree that the outcome ‘Genital pain during sexual activity ‘’ should be **included** in the Core Outcome Set?	Likert Scale	27% (n=3/11)	20% (n=2/10)	OUT
**8**	How much do you disagree or agree that the outcome ‘’Ability to achieve orgasm‘’ should be **included** in the Core Outcome Set?	Likert Scale	10% (n=1/10)	40% (n=4/10)	OUT

*Binary: [Disagree, Agree]; Likert Scale: [Strongly disagree, Disagree, Neither disagree nor agree, Agree, Strongly agree]
